# Spontaneous appendicocutaneous fistula in a child: a case report

**DOI:** 10.1093/jscr/rjag183

**Published:** 2026-03-22

**Authors:** Ayman A Albaghdady, Ahmed A Albaghdady, Amani N Alansari

**Affiliations:** Department of Pediatric Surgery, Faculty of Medicine, Ain Shams University, 61 Dr. AbdelShafy Mohamed St., 7th Zone, Nasr City, Cairo 11765, Egypt; Faculty of Medicine, Badr University in Cairo, Entertainment Area, Badr City, Cairo 11829, Egypt; Faculty of Medicine, Badr University in Cairo, Entertainment Area, Badr City, Cairo 11829, Egypt; Department of Pediatric Surgery, Hamad Medical Hospital, Zone 37, Street 222, PO Box 3050, Doha, Qatar

**Keywords:** appendicular fistula, appendicitis, cutaneous fistula, fistulogram

## Abstract

Spontaneous appendicocutaneous fistula is an extremely uncommon complication of acute appendicitis. We report a 6-year-old boy who developed a persistent feculent discharge from the right flank over the last 2 years after an inadequately treated case of appendicitis. Communication with the cecum was confirmed by diagnostic evaluation with fistulography. Appendectomy with complete excision of the fistulous tract resulted in full recovery.

## Introduction

Appendicular fistulas are very rare form of enteric fistula [1]. An appendiceal fistula results from spontaneous perforation of the appendix into an adjacent hollow viscus, such as the urinary bladder or bowel or, more rarely, into the abdominal wall or skin, making diagnosis particularly challenging [2, 3]. An appendiceal fistula arises from the spontaneous perforation of the appendix into an adjacent hollow organ, such as the urinary bladder or bowel. More rarely, it may perforate into the abdominal wall or skin, complicating the diagnosis significantly [4, 5]. Clinically, many cases evolve from a localized abscess that ruptures externally, leaving a chronically discharging sinus. Exploratory laparotomy is still the definitive diagnostic and therapeutic modality that allows appendectomy with excision of the tract [6].

## Case report

A 6-year-old boy presented with a two-year history of intermittently discharging right-flank sinus with occasional feculent and purulent output. Two years earlier, he developed right-lower-quadrant abdominal pain accompanied by fever and vomiting and was treated at a local facility with intravenous antibiotics. The family lived in a rural area with limited access to surgical services and follow-up and the child was also inadequately treated by the primary physician. Therefore, definitive surgical evaluation was delayed. During the current course, the child initially developed a tender swelling in the right flank that ruptured spontaneously, discharging pus and subsequently evolving into a persistently draining cutaneous opening. Following rupture, associated symptoms such as reduced appetite, abdominal fullness, nausea, and low-grade fever improved, but the flank opening continued to discharge intermittently, prompting referral to our service.

**Figure 1:**
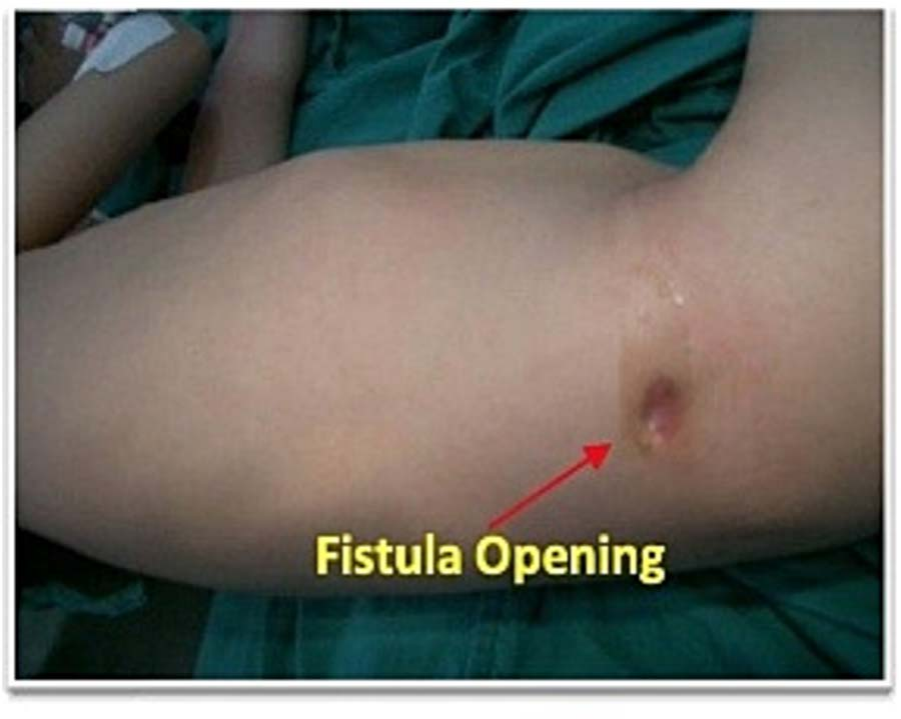
Preoperative photograph showing a right flank appendicocutaneous fecal fistula.

**Figure 2:**
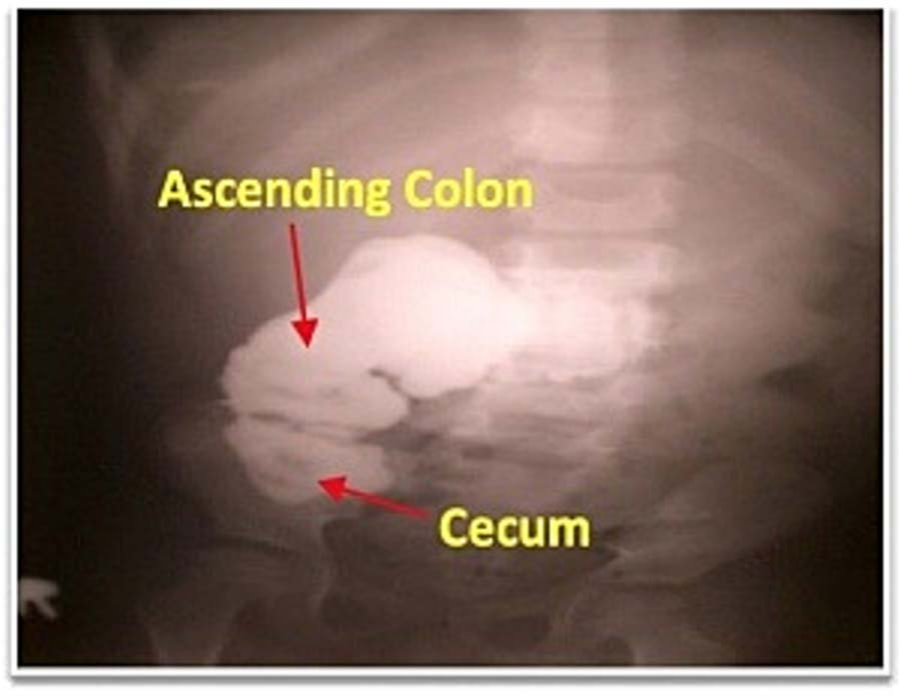
Fistulogram demonstrating contrast entering the cecum and right colon.

Abdominal examination revealed 1 cm of cutaneous opening at right lateral abdominal wall with intermittent production of pus-fecal material (Fig. 1). Laboratory evaluation showed a white blood cell count of 11 000/mm^3^ and an erythrocyte sedimentation rate of 7 mm/h, with all other hematologic, biochemical, and urine tests within normal limits. The chest radiograph was unremarkable. Pelvic radiography suggested superficial cortical irregularity of the right iliac bone, but no osteomyelitis. Clinically, there were no features of osteomyelitis such as: no localized bony tenderness or systemic toxicity and intraoperatively there was no gross bony sequestrum or purulent involvement requiring debridement. Fistulography performed through the flank opening delineated an irregular tract communicating with the cecum and extending along the ascending colon to the hepatic flexure (Fig. 2).

**Figure 3:**
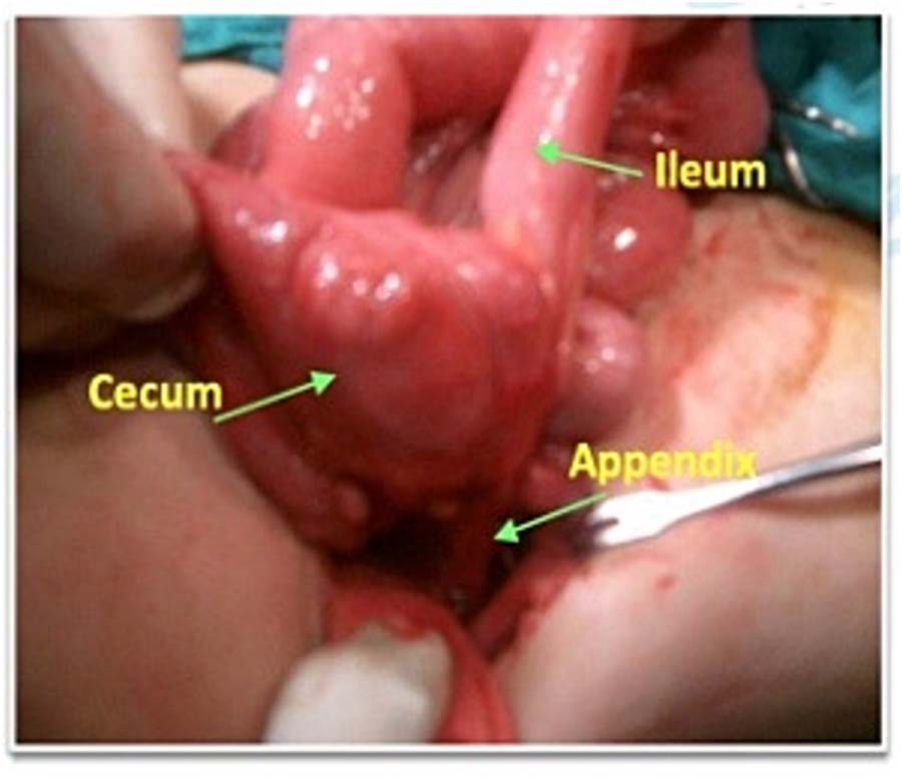
Intraoperative photograph showing the appendix traversing the fistula tract.

After the sinus discharge subsided, surgical exploration under general anesthesia was undertaken. Dense adhesions were noted in the ileocecal region, formed by an intestinal loop. The cecum and terminal ileum were identified, attached to the abdominal wall with a fibrous process (Fig. 3). The stump was then released using blunt and sharp dissection. A fistula tract was identified on the lateral wall of the inner face of the iliac bone, retroperitoneum at the level of the mid-axillary line. The remainder of the bowel appeared normal. An appendectomy with complete excision of the fistulous tract was performed. The wound was closed using 5–0 Vicryl in a subcuticular fashion. The patient received perioperative broad-spectrum antibiotics targeting enteric flora and the postoperative course was uncomplicated. So, he was discharged on postoperative day 5.

Histopathology revealed a fibrous tract lined with granulation tissue communicating with an appendix exhibiting acute and chronic inflammation, focal foamy aggregates, subserosal lymphocytic infiltration, and small ulcerations without granulomas. At 8-month follow-up, the child remained well.

## Discussion

Spontaneous appendicocutaneous fistula is a rare complication of inadequately treated appendicitis [7]. It typically forms following spontaneous rupture of an inflamed appendix, or may persist due to appendiceal calculi, carcinoid tumor, or tuberculosis [2, 8]. The cutaneous opening can occur at variable sites depending on the appendix’s position, most commonly on the anterior abdominal wall in the right lower quadrant, though presentations on the right flank or buttock have been reported [9, 10].

Most fistulas initially appear as subcutaneous abscesses, which, after rupture or drainage, may persist as nonhealing sinuses. These often discharge minimal pus or mucin, sometimes obscuring communication with the bowel on fistulography or barium studies [11]. Rarely, the tract is large, resulting in a fecal fistula with clear cecal communication on imaging, as observed in the present case. Plain abdominal radiographs may reveal appendicoliths, which can aid diagnosis when bowel communication is not evident [12]. Notably, inflammatory markers were not elevated (ESR 7 mm/h) despite the prolonged history, which may reflect a chronic, intermittently draining localized process without ongoing systemic inflammatory response.

Appendicocutaneous fistula is exceptionally uncommon in children, with pediatric cases reported sporadically in the literature, including: a 9-year-old boy with a retrocecal abscess tracking to the psoas presenting as a right-flank sinus, and a 12-year-old girl with cystic fibrosis presenting with a chronic right-iliac-fossa sinus [13, 14]. While all these reports describe a cutaneous tract from the appendix, our case includes a prolonged 2-year intermittent discharge and detailed imaging correlation that extend current understanding of the natural history and diagnosis of this uncommon complication. To the best of our knowledge, this patient is among the youngest reported children with a spontaneous appendicocutaneous fistula presenting as a right-flank opening. Early appendectomy with complete excision of the fistulous tract remains the recommended treatment, providing definitive resolution and preventing recurrence [15].

## Conclusion

Spontaneous appendicocutaneous fistula is an extremely rare complication of appendicitis that poses a diagnostic challenge. Fistulography is invaluable for establishing an accurate preoperative diagnosis, and early appendectomy with complete excision of the fistulous tract is the definitive treatment.
